# Investigating enhanced mechanical properties in dual-phase Fe-Ga-Tb alloys

**DOI:** 10.1038/srep34258

**Published:** 2016-10-03

**Authors:** Chongzheng Meng, Hui Wang, Yuye Wu, Jinghua Liu, Chengbao Jiang

**Affiliations:** 1 Key Laboratory of Aerospace Materials and Performance (Ministry of Education), School of Materials Science and Engineering, Beihang University, Beijing 100191, P. R. China

## Abstract

Dual-phase (Fe_83_Ga_17_)_100−*x*_Tb_*x*_ alloys with 0 ≤ *x* ≤ 1 were synthesized by arc melting and homogenization treatment. The microstructures and the corresponding mechanical properties were systematically investigated. The chemical composition of the body centered cubic matrix is Fe_83_Ga_17_. The monoclinic second phase was composed of meltable precipitates with approximate composition Fe_57_Ga_33_Tb_10_. The nano-hardness of matrix and precipitates were 2.55 ± 0.17 GPa and 6.81 ± 1.03 GPa, respectively. Both the ultimate tensile strength (UTS) and fracture strain (*ε*) of the alloys were improved by the precipitates for *x* ≤ 0.2 alloys, but the strain decreases significantly at higher values of *x*. As potential structural-functional materials, the best mechanical properties obtained were a UTS of 595 ± 10 MPa and an *ε* of 3.5 ± 0.1%, four-fold and seven-fold improvements compared with the un-doped alloy. The mechanism for these anomalous changes of mechanical properties was attributed to the dispersed precipitates and semi-coherent interfaces, which serve as strong obstacles to dislocation motion and reduce the stress concentration at the grain boundaries. A sizeable improvement of magnetostriction induced by the precipitates in the range 0 ≤ *x* ≤ 0.2 was discovered and an optimal value of 150 ± 5 ppm is found, over three times higher than that of the un-doped alloy.

Magnetostrictive materials are poised to play an increasingly important role in applications ranging from active vibration control and energy harvesting to stress and torque sensing^1^. One of the most famous materials is the Tb-Dy-Fe alloy (Terfenol-D) which shows a magnetostriction at ambient temperature which may be as large as 2000 ppm^2^. However, the disadvantages of a high magnetic field needed for saturation, mechanical brittleness and the need of expensive heavy rare earth elements has inspired a wave of interdisciplinary efforts aimed at finding more sustainable high-performance magnetostrictive materials. As the most promising candidate, Fe-Ga (Galfenol) single crystals combine certain advantages such as low saturation magnetic field (100 Oe), moderate tetragonal magnetostriction (*λ*_001_ = 280 ppm), ductility and lower cost^3^,^4^,^5^,^6^. Compared with traditional functional materials such as Terfenol-D and piezoelectric ceramics, the better mechanical properties of Galfenol make it attractive for practical applications under harsh conditions^7^. As a consequence, over last decades Galfenol alloys have been the research focus for novel magnetostrictive materials with excellent functional and structural properties.

Admittedly, the relatively low intrinsic magnetostriction and inherent brittleness in polycrystalline Fe-Ga alloys, confirmed by previous works^8^,^9^ are a drawback; but micro-alloying is an effective way to solve these problems. Firstly, it is believed that the strong magnetostrictive responses of Fe-Ga alloys result from local tetragonal distortion of the matrix which is triggered by a heterogeneous nanostructure with coherent tetragonal nano-inclusions^3^,^6^,^10^,^11^. Expansion of the tetragonal distortion is the major route to improve the magnetostriction of these materials. Great efforts have been made following this idea by adding 3*d* and 4*d* transition elements such as V, Cr, Mn, Co, Ni, Cu, Zn, Nb, Mo and Rh^12^,^13^,^14^,^15^,^16^; main group elements such as Be, C, B, N, Si, Al, Ge, Sn^17^,^18^,^19^,^20^,^21^,^22^; and rare earth elements such as Y, La, Ce, Tb, Dy…^11^,^23^,^24^,^25^,^26^,^27^,^28^,^29^. Trace doping with rare earth elements has been confirmed to be the most effective way to influence the induced tetragonality of the matrix and it greatly enhances the magnetostriction of the Fe-Ga alloys^11^. Secondly, a body of work has been performed to optimize the mechanical properties of Fe-Ga through addition of the third elements such as B, C, Al, V, Cr, Mo, Mn and Nb^13^,^16^,^17^,^18^,^22^,^30^,^31^, or refractory carbide precipitates such as X–C (X = Zr, Nb, Ta, Mo)^32^,^33^,^34^,^35^,^36^. As a result, effective improvements of mechanical properties have been obtained by means of the alloy additions or the formation of carbide precipitates, but there is no significant improvement of the magnetostriction. Fe-Ga based alloys which combined excellent mechanical properties with enhanced magnetostriction are currently unavailable.

Here we report novel Tb doped Fe-Ga alloys exhibiting an excellent magnetostriction that is combined with improved mechanical properties. In order to explore the usefulness and tailor the properties of the new Fe-Ga-Tb alloys; we investigate the mechanical properties at ambient temperature systemically and analyze how the semi-coherent interfaces toughen the materials during tensile deformation. Compared with the un-doped binary Fe-Ga alloys, both ultimate tensile stresses and strains were significantly enhanced due to the precipitation of Tb-rich phases. Large ultimate tensile strength of 595 ± 10 MPa and fracture strain of 3.5 ± 0.1% were obtained in alloys with 0.2 at.% Tb addition. Sizeable improvement of magnetostriction induced by the precipitates in the range 0 ≤ *x* ≤ 0.2 was discovered, and the optimal value of 150 ± 5 ppm is three times higher than that of the as-cast polycrystalline un-doped alloy.

## Results and Discussions

**Figure 1:**
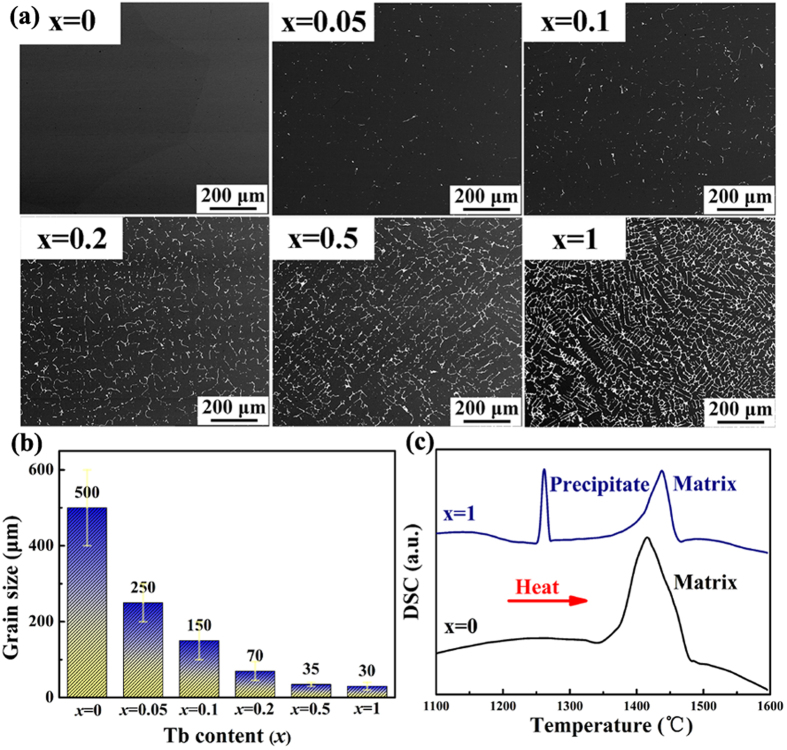
Observations and thermal analysis of dual-phase Fe-Ga-Tb alloys with different Tb addition. (**a**) Back scattered electron images and (**b**) Tb content dependence of grain size of (Fe_0.83_Ga_0.17_)_100−*x*_Tb_*x*_ alloys with *x* = 0, 0.05, 0.1, 0.2, 0.5, 1; (**c**) DSC curves of *x* = 0 and *x* = 1 specimens.

Figure 1(a) displays typical back scattered electron (BSE) images of (Fe_0.83_Ga_0.17_)_100−*x*_Tb_*x*_ alloys with 0 ≤ *x* ≤ 1. In the un-doped binary alloy (*x* = 0), single-phase morphology with coarse equiaxed grains is observed with an average grain size of ~500 μm. In the Tb-doped ternary alloys, bright spheres and rods are observed which prove that a dual-phase structure of matrix and precipitates is obtained. The volume fraction of the precipitates increases with Tb content and the distribution mode changes from a dispersion to a continuous network. Energy dispersive spectroscopy (EDS) is adopted to analyze the chemical composition of both phases, the precipitates (Fe 56.62 at.%, Ga 33.28 at.%, Tb 10.10 at.%) are confirmed to be of a Tb-rich phase compared with the matrix (Fe 83.44 at.%, Ga 16.56 at.%), as shown in Table 1. Figure 1(b) is a histogram relating the matrix grain size and Tb content. Obviously, the Tb addition results in a remarkable grain refinement of the Fe-Ga from 250 μm for *x* = 0.05 to 30 μm for *x* = 1. For the alloy with *x* = 0.05, homogeneous equiaxed grains with straight boundaries are observed. When *x* was increased to 1, the refined grains show a quite different morphology. Dendrites appears with destabilized and winding grain boundaries. Besides, it is clear that a secondary phase exists at the grain boundaries. DSC measurements were performed to better understand the formation of the dual-phase morphologies for *x* = 0 and *x* = 1 specimens in the temperature range 1100–1600 °C, as shown in Fig. 1(c). The melting points of matrix and precipitates are approximately 1450 °C and 1250 °C. The meltable precipitates significantly change the solidification behavior of matrix; Fe_83_Ga_17_ first solidified from the solid-liquid interface to form the dendrite skeleton in the primary period of solidification. Later, the interdendritic segregation of Tb-rich phases precipitated on decreasing temperature, which divides the matrix into many dispersed regions.

**Figure 2:**
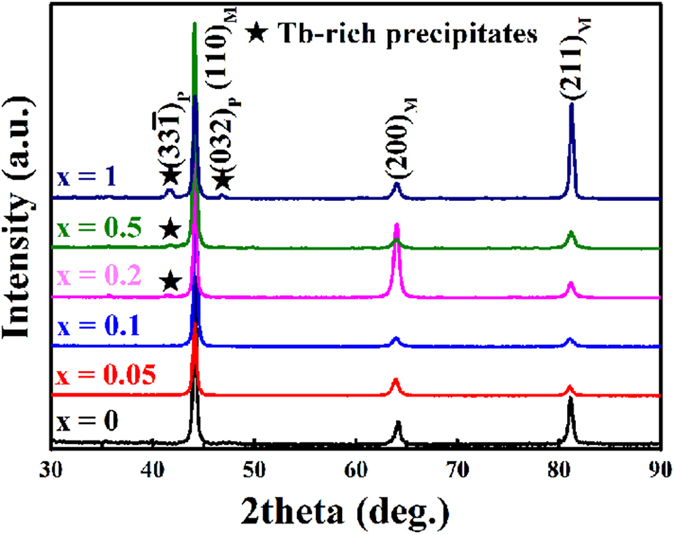
XRD patterns of (Fe_0.83_Ga_0.17_)_100−*x*_Tb_*x*_ (0 ≤ *x* ≤ 1) alloys with different Tb addition.

XRD patterns are carried out at ambient temperature to understand the phase structures of the alloys studied, as shown in Fig. 2. All the main diffraction peaks are indexed on a body centered cubic structure, which illustrates that the matrix possesses the same crystal structure as the un-doped binary alloy. For Tb-doped ternary alloys with *x* ≥ 0.2, some unknown diffraction peaks at 2*θ* ≈ 41.6° and 46.8° marked by stars are thought to arise from the Tb-rich precipitates. Overall, the XRD results are consistent with the BSE morphologies. Further investigations of the crystal structure of the precipitates are discussed latter.

**Figure 3:**
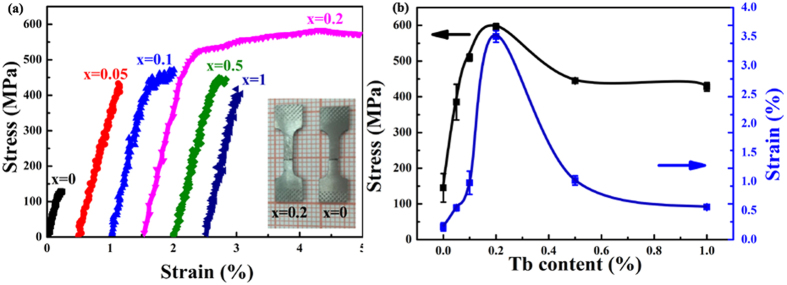
Mechanical properties of (Fe_0.83_Ga_0.17_)_100−*x*_Tb_*x*_ (0 ≤ *x* ≤ 1) alloys with different Tb addition. (**a**) Tensile stress-strain curves of (Fe_0.83_Ga_0.17_)_100−*x*_Tb_*x*_ (0 ≤ *x* ≤ 1) alloys (Inset: the fracture specimens of *x* = 0 and *x* = 0.2); (**b**) Tb content dependence of ultimate tensile strength and fracture strain for (Fe_0.83_Ga_0.17_)_100−*x*_Tb_*x*_ (0 ≤ *x* ≤ 1) alloys.

With the aim of exploring the effect of Tb-rich precipitates on the mechanical properties, the tensile stress-strain curves were measured on many specimens of each alloy at ambient temperature, as represented in Fig. 3(a). The dependence of ultimate tensile strength and fracture strain on Tb content is summarized in Fig. 3(b). The detailed mechanical properties of the (Fe_0.81_Ga_0.19_)_100−*x*_Tb_*x*_ (0 ≤ *x* ≤ 1) alloys are displayed in Table 2. Notably, two features can be extracted. Firstly, the ultimate tensile strengths and fracture strains are dramatically improved with the formation of precipitates in the range of *x* < 0.2, but the strain significantly decreases while the strength remains relatively stable with greater *x* and a higher precipitate fraction. For the un-doped binary alloy, typical elastic deformation is shown in the stress-strain curve. The ultimate tensile strength is 145 ± 40 MPa and the corresponding fracture strain is 0.22 ± 0.07%, which is close to previous results^32^. With the addition of Tb, both the tensile strength and fracture strain increase rapidly and reach the maximum values of 595 ± 10 MPa and 3.5 ± 0.1% in the *x* = 0.2 specimen. When the Tb content shifts to a higher value, the tensile strength gradually decreases to 428 ± 12 MPa while the fracture strain falls sharply to 0.56 ± 0.02% for *x* = 1. Secondly, the yield behavior observed in the tensile stress-strain curves of *x* = 0.1, 0.2 and 0.5 specimens, indicates the appearance of partial ductile deformation in these alloys. Especially in the *x* = 0.2 alloy, the yield behavior provides the large ultimate tensile strain of 3.5 ± 0.1%, and this performance is almost comparable to the reported highest ambient temperature tensile strain in Fe-Ga based alloys^13^,^16^,^17^,^18^,^22^,^30^,^31^. From the ambient temperature tensile stress-strain measurements of the (Fe_0.83_Ga_0.17_)_100−*x*_Tb_*x*_ alloys, it is apparent that a very small amount of Tb-rich precipitates can dramatically enhance the mechanical properties and even induce yield behavior beyond the elastic limit with 0 < *x* ≤ 0.2, while excess addition of Tb leads to weakening of the ductility with 0.2 < *x* ≤ 1. In the light of these results, more microscopic analysis in needed to explain the evolution of the mechanical behaviors with increasing Tb content in (Fe_0.83_Ga_0.17_)_100−*x*_Tb_*x*_ system.

**Figure 4:**
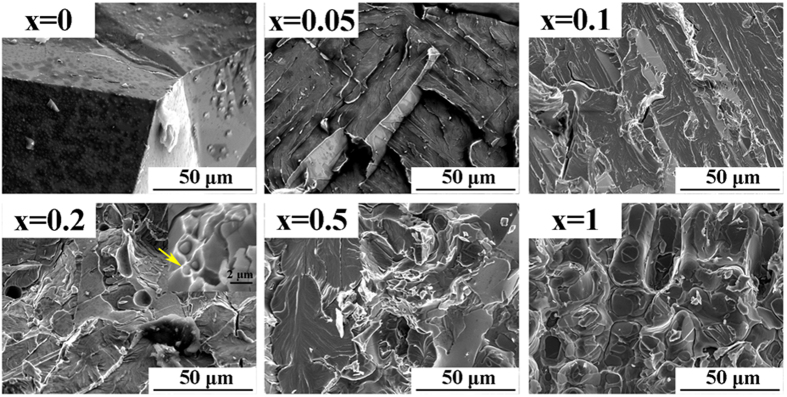
SEM observations show the fracture morphologies of (Fe_0.83_Ga_0.17_)_100−*x*_Tb_*x*_ (0 ≤ *x* ≤ 1) alloys with different Tb addition (Inset: the magnified fracture image).

Figure 4 shows representative fracture surfaces of (Fe_0.83_Ga_0.17_)_100−*x*_Tb_*x*_ alloys with 0 ≤ *x* ≤ 1 observed by SEM. For the un-doped binary alloy, a typical brittle fracture character with a smooth surface of coarse grains can be observed, which proves that intergranular fracture accompanies the severe deterioration in ductility. This is consistent with the result of low tensile strength and strain detected in Fig. 3(a). The fracture surface gradually becomes rough with a slight addition of Tb, *x* = 0.05, and further exhibits a river pattern which indicates an apparent transgranular cleavage characteristic for *x* = 0.1. For the *x* = 0.2 alloy, the one with the best mechanical properties, not only is the morphology rough but a small amount of dimples with circular shape can be discovered (Inset of *x* = 0.2 image). EDS is used to detect the chemical compositions of the regions in and around the dimples, which confirms that the center of the dimples is Tb-rich. This evidence suggests that the discontinuously distributed precipitates make a contribution to the appearance of plastic deformation, so the ductility is remarkably improved. When the Tb content switches to a higher value, smooth areas with fragments emerge in the fracture surface of the *x* = 0.5 specimen and plenty of fragments cling to the surface of the grains in the fracture morphology for the *x* = 1 specimen. In view of the BSE morphologies in Fig. 1(a), the fragments should be the continuously distributed Tb-rich precipitates. It is observed that the alloys fracture along the precipitate/matrix interfaces in specimens with high Tb addition (*x* = 0.5, 1), which indicates that interphase fracture becomes the predominant fracture mode. Nanoindentation measurement was employed to further study the mechanical properties of the precipitates in comparison with the matrix, and the results are exhibited in Table 3. Obviously, a prominent disparity of nano-hardness between the matrix and the precipitates is identified, with respective values of 2.55 ± 0.17 GPa and 6.81 ± 1.03 GPa. Based on the nanoindentation result, the precipitates are considered to be hard and brittle compared with the matrix. As a result, continuous precipitates provide an unrestricted path for the extension of cracks in specimens with excessive Tb addition, and the fracture morphologies reflect the path of continuous precipitates and precipitate/matrix interfaces^37^. Hence the poor mechanical character of the precipitates hinders further improvement of the ductility in Fe-Ga-Tb alloys.

To study mechanical properties, we generally focus on the two major characteristics of ultimate tensile strength and fracture strain, which embody the strength and the ductility of alloys, respectively. It can be quite accurate to think the origin of the excellent mechanical properties in Fe-Ga-Tb alloys is grain refinement and the transformation of the fracture mode. Firstly, the dependence of the ultimate tensile strength on the grain size of single-phase metals has fascinated researchers ever since Hall^38^ and Petch^39^ discovered the inverse dependence of strength on grain size. The corresponding relation is known as the Hall-Petch relationship, which can be expressed as σ_s_ = σ_0_ + *kd*^−1/2^. This formula is proposed on the basis of the dislocation pile-up model and mainly considers the interaction between dislocations and grain boundaries. In the conditions of our present work, the effect of grain refinement on the ultimate tensile strength in dual-phase systems can be explained some degree by the Hall-Petch relationship. For the alloys with 0 ≤ *x* ≤ 0.2, the grains are significantly refined from ~500 μm to ~150 μm, and the volume fraction of Tb-precipitates remains small. In these conditions, the grain refinement effect efficiently hampers the movement of dislocations, and leads to the fracture mode transformation from intergranular to transgranular. Therefore, grain refinement plays a key role in the improvement of the ultimate tensile strength. The UTS remains relatively stable with increasing fraction of Tb-rich precipitates and finer grains, which is in disagreement with the Hall-Petch relationship. According to the fracture surface morphologies in Fig. 4, few transgranular fracture regions can be found for the alloys with 0.2 < *x* ≤ 1; instead the alloys fracture along the precipitate/matrix interfaces when the Tb content has a higher value. The fracture morphologies signify that crack initiation and propagation maybe occur in the precipitates on account of their poor mechanical properties identified by the nanoindentation measurement. So the continued grain refinement has a weak influence on ultimate tensile strength, and UTS is mainly determined by the fracture mechanism.

Secondly, the cause of an outstanding *ε* is even more complex. The dependence of ductility on decreasing grain size presents a unimodal variation. For the specimens with *x* = 0, 0.05 and 1, each tensile stress-strain curve only exhibits an elastic segment. While for the remaining specimens, the tensile stress-strain curves are composed by two segments, one elastic and the other plastic. The family of tensile curves clearly exhibits characteristics changing from brittleness to ductility and back again to brittleness with the continuous grain refinement. Moreover, Young’s modulus and stress of the elastic limit for the specimens with different Tb content are almost the same for them all. Therefore, a small amount of Tb-rich precipitates can dramatically enhance the mechanical properties and even induce plastic yield behavior, while excess addition of Tb leads to a lessening of the ductility. Based on the above evidence, it is considered that the grain size effect directly improves the tensile stress and indirectly influences the fracture strain of the elastically deformed segments. However, the appearance and disappearance of plastic deformation cannot be explained by the grain size effect. Therefore, more microstructural information is still essential to understand the specific ductility behavior in Fe-Ga-Tb alloys.

**Figure 5:**
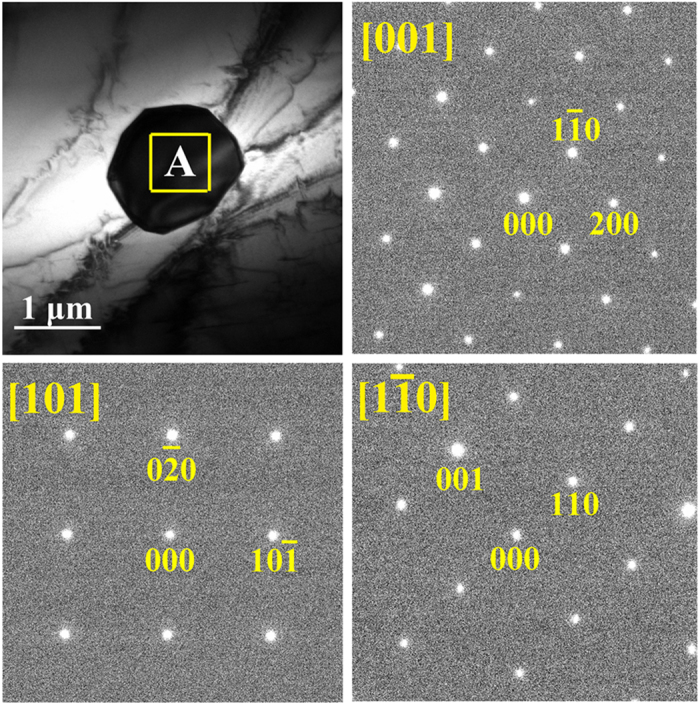
TEM morphology of the (Fe_0.83_Ga_0.17_)_99.8_Tb_0.2_ alloy and SAED patterns taken from area A with the incident electron beam tilted to [001], [101] and [1
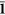
0], respectively.

Figure 5 shows the morphology and corresponding selected area electron diffraction (SAED) of Tb-rich precipitate in the *x* = 0.2 alloy, which can be indexed as [001], [101] and [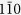] zone axes of a monoclinic structure with lattice parameters *a* = 1.044 nm, *b* = 0.902 nm, *c* = 0.524 nm and *β* = 103.4°. In the other dual-phase alloys, the crystal structure of the precipitates is the same. With the assistance of TEM analysis, the additional X-ray diffraction peaks for *x* ≥ 0.2 alloys in Fig. 2 could be indexed as 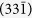 and (032). Furthermore, the precipitate/matrix interface structure in the *x* = 0.2 alloy was investigated to explore the ductility mechanism of the alloys by TEM analysis.

**Figure 6:**
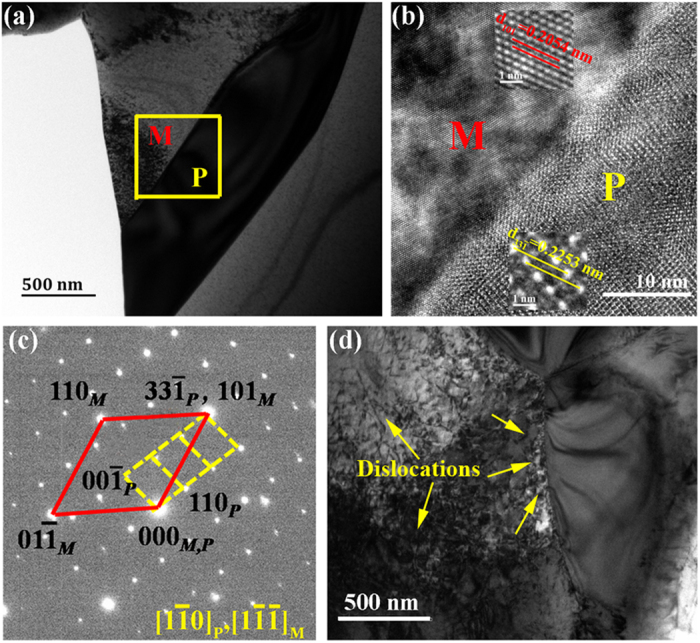
TEM observations of the semi-coherent interface and dislocation configurations found in a (Fe_0.83_Ga_0.17_)_99.8_Tb_0.2_ alloy. (**a**) BF image of the (Fe_0.83_Ga_0.17_)_99.8_Tb_0.2_ alloy and (**b**) corresponding HREM pattern taken from the yellow box; (**c**) SAED patterns across the interface between the precipitate and the matrix; (**d**) Microstructure evolution near the fracture section.

Figure 6(a) shows a representative bright-field (BF) image of the *x* = 0.2 specimen which provides evidence that the grains are dislocation-free before the tensile tests. High resolution transmission electron microscopy (HRTEM) and selected area electron diffraction (SAED) were carried out in the selected area marked by the yellow box that included the precipitate/matrix interface, as shown in Fig. 6(b,c). It is clear that the precipitate prefers a semi-coherent relationship with the matrix, where the (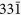) plane of the precipitate is parallel to the (101) plane of the matrix, i.e. (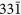) _P_//(101)_M_ with the interplanar spacing of 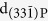 = 0.2253 nm and d_(101)M_ = 0.2054 nm, respectively. The interplanar spacing of the precipitate is 9.7% larger than the spacing of the corresponding planes in the matrix, so local expansion occurs along the [101] direction in the matrix. Meanwhile, the zone axis direction of the [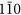] orientation of the precipitate is parallel to the [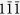] orientation of the matrix, i.e. [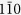]_P_//[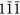]_M_. As a consequence, it can be concluded that the presence of such semi-coherent precipitate/matrix interfaces just induce anomalous lattice distortions, which cause a local elastic strain field to absorb and pin dislocation movement during alloy deformation^40^,^41^,^42^. Figure 6(d) shows the BF image of a fractured specimen taken from the position near the fracture section for the *x* = 0.2 specimen. It can be seen that the precipitate is surrounded by high-density dislocations accompanied by a remarkable contrast of stress field, which reflects the interactions between precipitates and dislocations.

According to the TEM research, the anomalous regularity of the fracture strain with different Tb additions should be directly correlated with the storage ability of dislocations in the specimen. In single-phase metals and alloys, the type of dislocation is mainly statistically stored dislocations (SSD). However, in dual-phase alloys with micron scale particles, the heterogeneous deformation rate between the different phases produces a significant strain gradient, the dislocations are generally induced to coordinate the misfit deformation. This type of dislocation is defined as a geometrically necessary dislocation (GND)^43^,^44^,^45^. In the *x* = 0.2 alloy, an appropriate particle size of 1–2 μm and weak deformability of the precipitates provide favorable conditions for the appearance of large amounts of GNDs around the semi-coherent interfaces during the initial stage of plastic deformation, while the subsequent existence and accumulation of massive SSDs lead to further increase of the dislocation densities. Meanwhile, the dispersed particles forming the semi-coherent interface lead to a quite strong combined strength of the matrix with the precipitates that serve as obstacles to dislocation motion and reduce stress concentration on the grain boundary, which postpones the onset of geometric instabilities to higher strains. In this situation, the dispersed Tb-rich precipitates and semi-coherent interfaces introduce very high dislocation densities, which results in the yield behavior and large tensile strains on the macro-scale.

**Figure 7:**
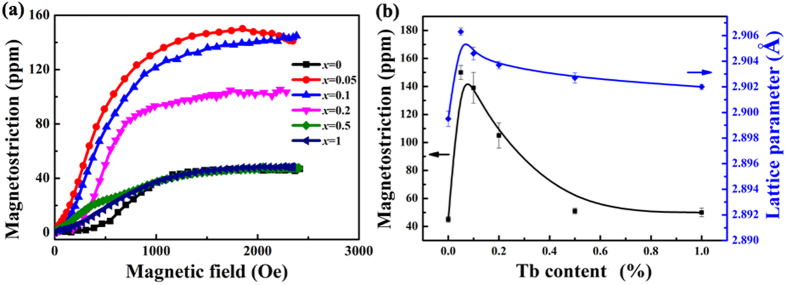
Measured magnetostriction of (Fe_0.83_Ga_0.17_)_100−*x*_Tb_*x*_ (0 ≤ *x* ≤ 1) alloys with different Tb additions. (**a**) Measured magnetostriction curve of (Fe_0.83_Ga_0.17_)_100−*x*_Tb_*x*_ (0 ≤ *x* ≤ 1) alloys; (**b**) Tb content dependence of magnetostriction and lattice parameter for (Fe_0.83_Ga_0.17_)_100−*x*_Tb_*x*_ (0 ≤ *x* ≤ 1) alloys.

Particularly worth mentioning in view of the potential structural-functional integration of Fe-Ga-Tb alloys is the dependence of the magnetostriction on Tb content. Figure 7(a) shows the measured magnetostriction of the as-cast (Fe_0.83_Ga_0.17_)_100−*x*_Tb_*x*_ alloys. For the binary Fe-Ga alloy, the magnetostriction is only 45 ± 2 ppm, which is in good agreement with previous reports^33^. A great increase of the magnetostriction of ternary alloys has been induced by doping with very small amounts of Tb. The magnetostriction of the *x* = 0.05 specimen is as high as 150 ± 5 ppm, which is triple that of the un-doped alloy. However, there is a similar monotonic decrease of the magnetostriction and the lattice parameter with increased Tb content. The magnetostriction of the *x* = 0.2 specimen drops to 105 ± 9 ppm, still more than double that of the un-doped alloy. It declines continuously to 50 ± 3 ppm in the *x* = 1 specimen, which is approximately the same as the un-doped alloy. As we know, the magnetostriction of an alloy is determined by the lattice distortion and local strain field, and trace doping Tb will introduce a larger lattice distortion and obviously enhance the magnetostriction^3^. Therefore, the *x* dependence of the magnetostriction and the lattice parameter are both summarized in Fig. 7(b), where find that both exhibit a consistent overall tendency. It is reasonable to believe that the doping of Tb in Fe-Ga alloys gives rise to large lattice distortion in the matrix and induces a larger magnetostrictive effect in the range 0 ≤ *x* ≤ 0.2, while an increased fraction of Tb-rich precpitates has a negative effect on the magnetostrictive properties and leads to the significant reduction of the magnetostriction.

## Conclusion

A dual-phase microstructure is formed in (Fe_0.83_Ga_0.17_)_100−*x*_Tb_*x*_ alloys with 0 ≤ *x* ≤ 1, where the matrix retains the A2 structure, and the precipitates are found to possess a monoclinic structure. The precipitates possess a lower melting point (1250 °C) and superior hardness (6.81 ± 1.03 GPa) compared with the matrix, which greatly influences the mechanical properties. The influence of Tb on the mechanical properties in (Fe_0.83_Ga_0.17_)_100−*x*_Tb_*x*_ (0 ≤ *x* ≤ 1) is most evident in the tensile measurements. Both ultimate tensile strengths and fracture strains are initially improved by the monoclinic Tb-containing precipitates for alloys with *x* ≤ 0.2, but the fracture strain significantly decreases with the higher fraction of precipitates. The best mechanical properties were obtained in the *x* = 0.2 alloy, with an ultimate tensile strength of 595 ± 10 MPa and fracture strain of 3.5 ± 0.1%, much enhanced compared with the un-doped binary Fe-Ga alloy, respectively. The transformation of the fracture mode in the alloys from intergranular fracture to transgranular fracture and subsequently to interphase fracture with increasing Tb addition was revealed to be responsible for the evolution of the mechanical performance. Dispersed Tb-rich precipitates and semi-coherent interfaces introduce extremely high dislocation densities, which results in excellent yield behavior and large macro-scale tensile strains for the *x* = 0.2 alloy. Sizeable improvement of magnetostriction induced by the precipitates in the range 0 ≤ *x* ≤ 0.2 was discovered and an optimal value of 150 ± 5 ppm is determined for the alloy with *x* = 0.05, which is over three times that of the un-doped alloy. The unusual composition of excellent mechanical properties and high magnetostriction can be expected to lead to many applications of this new functional alloy.

## Methods

High-purity starting elements iron, gallium and terbium were arc-melted for the preparation of (Fe_0.83_Ga_0.17_)_100−*x*_Tb_*x*_ (*x* = 0, 0.05, 0.1, 0.2, 0.5, 1) master alloys under argon atmosphere. Each ingot was melted four times to assure homogeneity. The ingots were annealed at 1100 °C for two hours and then furnace cooled to ambient temperature. The crystallographic structure was identified by x-ray diffraction (XRD, D/MAX 2200 PC) with Cu-K_α_ radiation (*λ* = 1.5418 Å and scanning speed of 6°/min). The chemical compositions were characterized by JEOL JXA-8100 electron probe micro-analyzer (EPMA) equipped with an energy dispersive spectrometer (EDS) analysis system. Ambient temperature tensile test was carried out on a computer controlled Instron-8801 testing machine with a constant stain rate of 1 × 10^−4^ s^−1^. Each tensile specimen with a gauge length of 10 mm was cut from the center of ingots by an electric discharge wire saw. Scanning electron microscopy (SEM, JMS-6010) was used to investigate the morphologies, phase distribution and fracture surfaces of the fractured specimen. The melting point of (Fe_0.83_Ga_0.17_)_100−*x*_Tb_*x*_ alloys were determined by LABSYS-1600 differential scanning calorimetry (DSC) at a heating rate of 10 °C/min. Magnetostriction was measured by the standard strain gauge method. Nanoindentation measurements were performed at room temperature on the surface of the specimens using an Agilent Technologies XP nanoindenter (Agilent Technologies Co., Nano Instruments, Oak Ridge, TN, USA) equipped with a Berkovich (three-sided pyramidal) diamond tip. To reduce effects of specimen preparation and surface roughness, a large indentation load of 1000 mN was used. For each phase, 5 indents were duplicated to obtain reliable indentation data. The indents separated as far as much as possible to avoid the boundary effect. For all indents, the same loading/unloading rate (10 mN/s) and holding time at the maximum indentation load (30 s) were used.

## Additional Information

**How to cite this article**: Meng, C. *et al.* Investigating enhanced mechanical properties in dual-phase Fe-Ga-Tb alloys. *Sci. Rep.* **6**, 34258; doi: 10.1038/srep34258 (2016).

## Figures and Tables

**Table 1:** EDS results of dual-phase Fe-Ga-Tb alloys.

In alloy	Element	wt.%	at.%
Matrix	Fe	80.15	83.44
	Ga	19.85	16.56
Precipitates	Fe	44.62	56.62
	Ga	32.74	33.28
	Tb	22.64	10.10

**Table 2:** Mechanical properties of the (Fe_0.81_Ga_0.19_)_100−*x*_Tb_*x*_ (0 ≤ *x* ≤ 1) alloys.

No.	In alloy	UTS, MPa	ɛ, %
1	*x* = 0	145 ± 40	0.22 ± 0.07
2	*x* = 0.05	385 ± 50	0.55 ± 0.05
3	*x* = 0.1	510 ± 10	0.98 ± 0.2
4	*x* = 0.2	595 ± 10	3.5 ± 0.1
5	*x* = 0.5	445 ± 6	1.02 ± 0.08
6	*x* = 1.0	428 ± 12	0.56 ± 0.02

**Table 3:** Nanoindentation results for dual-phase Fe-Ga-Tb alloys.

In alloy	E Average Over Defined Range (GPa)	H Average Over Defined Range (GPa)
Matrix	160.44 ± 2.91	2.55 ± 0.17
Precipitates	179.85 ± 10.06	6.81 ± 1.03
